# Pararectal Leiomyosarcoma Following Total Laparoscopic Hysterectomy (TLH) for Initially Diagnosed Uterine Leiomyoma

**DOI:** 10.1002/ccr3.73090

**Published:** 2026-11-04

**Authors:** Nanna Monjé, Matthias Alexa, Annette Hasenburg, Yaman Degirmenci

**Affiliations:** ^1^ Department of Gynecology and Obstetrics University Medical Center of the Johannes Gutenberg University Mainz Germany

**Keywords:** fibroids, hysterectomy, leiomyomas, Leiomyosarcoma, uterine neoplasms

## Abstract

This case report highlights the diagnostic challenges of clinically distinguishing leiomyomas and leiomyosarcomas and the importance of post‐hysterectomy monitoring. Patients with extensive prior oncological treatments and genetic cancer predisposition require individualized surveillance strategies, as they remain at elevated risk for secondary malignancies that may present in unusual locations or timeframes.

## Graphical abstract

Pelvic MRI one year post total laparoscopic hysterectomy (TLH) revealed suspicion of a pararectal and a paraurethral recurrence of the initially diagnosed uterine leiomyoma.

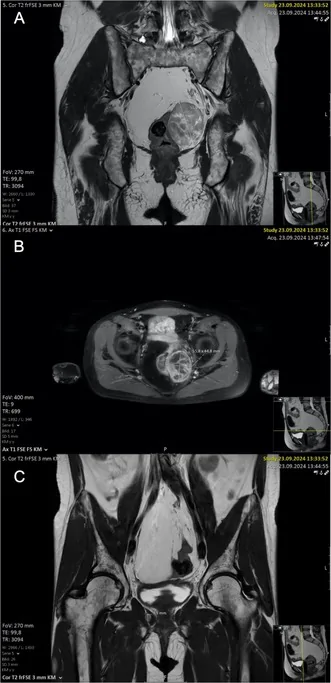

## 1 Introduction

Uterine leiomyomas are among the most common benign tumors of the female genital tract, affecting up to 50% of women of childbearing age [1]. Following hysterectomy for a uterus myomatosus, the risk of intrauterine fibroid recurrence is eliminated. However, rare cases of extrauterine so‐called parasitic leiomyomas following previous fibroid surgeries have been documented, particularly when morcellation techniques were employed [2, 3] but also after total transabdominal hysterectomy [4].

Diagnosing such cases is challenging because the clinical presentation is nonspecific and it is essential to consider sarcomas as a differential diagnosis. This presents a significant challenge in clinical practice because neither clinical examination nor standard imaging techniques (such as ultrasound, CT‐ or MRI‐scans) can reliably distinguish between these two conditions. However, magnetic resonance imaging (MRI) demonstrates well‐characterized features indicative of leiomyosarcoma and represents the imaging modality of choice for distinguishing benign leiomyomas from lesions with abnormal tissue metabolism [5]. In the final instance histopathological examination is crucial to establish a definitive diagnosis and differentiate between benign metastatic uterine fibroids and malignant tumors such as leiomyosarcoma (LMS) [6]. The annual incidence of uterine leiomyosarcoma is approximately 0.4 per 100,000 women, with the highest frequency observed between 45 and 59 years of age. Moleculary, the disease is characterized by alterations in the tumor suppressor genes p53 and Rb‐1, and about 10% of cases exhibit BRCA2 mutations [6]. Extrauterine LMS most commonly occur in the retroperitoneum and the extremities [7]. Preliminary exposure to chemotherapy or radiotherapy is associated with a heightened risk of the development of a soft tissue sarcoma. In cases where there is suspicion of a sarcoma developing as a secondary malignancy following prior cancer treatment, an underlying tumor predisposition should always be taken into account [8].

This report describes a rare case of an extrauterine leiomyosarcoma following total laparoscopic hysterectomy for a uterus myomatosus in a 47‐year‐old female diagnosed and treated at the Clinic for Obstetrics and Women's Health at the University Medical Center of the Johannes Gutenberg University Mainz, Germany. To our knowledge, this is the first case of a LMS diagnosed 1 year after an initial benign diagnosis in a patient who underwent hysterectomy without morcellation, highlighting its exceptional rarity. The aim of this case report is to highlight the diagnostic challenges and therapeutic approaches while raising awareness of this uncommon entity.

## 2 Case History

The patient was a 47‐year‐old woman (gravida II, para I) who first presented at our clinic 2 years ago. The referring gynecologist had noticed progressively enlarging fibroids. Additionally, the patient reported left‐sided pain during defecation. Transvaginal ultrasound at that time revealed multiple uterine fibroids. There was suspicion of a pedunculated anterior wall fibroid measuring 3.7 × 2.3 cm.

As a secondary diagnosis, the patient had multiple pre‐existing conditions. In childhood, she had acute lymphoblastic leukemia (first diagnosed in early childhood, with relapse 2 years after initial diagnosis), for which she had received multiple courses of anthracycline‐containing chemotherapy and had undergone bone marrow transplantation. During early adulthood, she was also diagnosed with thyroid carcinoma, for which she had undergone thyroidectomy and radioiodine therapy. Furthermore, she had known type II diabetes mellitus, arterial hypertension, and osteopenia. Genetic counseling had already been conducted previously due to additional gynecological tumor diseases in the family history, and a known BRCA1 mosaic was present.

Considering these findings and pre‐existing conditions, a total laparoscopic hysterectomy with bilateral adnexectomy was performed. Intraoperatively, an enlarged uterus due to anterior and lateral wall fibroids was observed, while the adnexa appeared unremarkable. The uterus was transvaginally removed in toto (colpotomy). The operation proceeded without complications, no morcellation was performed, and there was no evidence of extrauterine fibroid manifestations. Histopathology revealed several intramural fibroids, with the largest measuring 5.0 cm. Microscopically, these masses were composed of plump‐spindle cells with interwoven fascicles. There was no increased mitotic rate and no evidence of malignancy. The fallopian tubes were age‐appropriately unremarkable, while the ovaries showed bilateral adenofibromas.

About 1 year after the initial presentation transvaginal ultrasound for postoperative follow‐up revealed a 4 cm heterogeneous hyperechogenic finding in the pelvic wall area, which appeared to be a recurrence of a leiomyoma. A subsequently initiated MRI examination revealed suspicion of a pararectal and a paraurethral fibroid recurrence (Figure 1). In the lateral to dorsolateral left pararectal area of the pelvis, there was a 5.6 × 5.5 × 4.3 cm, smoothly bordered and T2 heterogeneous mass. Additionally, a lesion was found anteriorly at the level of the urethra and vagina between the levator ani muscle and the obturator internus muscle. This measured 11 × 7 × 9 mm, was smoothly bordered, and showed strong homogeneous contrast enhancement. The images were presented at a preoperative gynecological board in our clinic, where the MRI T2‐weighted images revealed bizarre contrast enhancement of the pararectal mass. However, there was a clear demarcation from the rectum. Therefore, the mass was still assumed to be a fibroid recurrence in the form of a parasitic fibroid and surgery was indicated.

**FIGURE 1:**
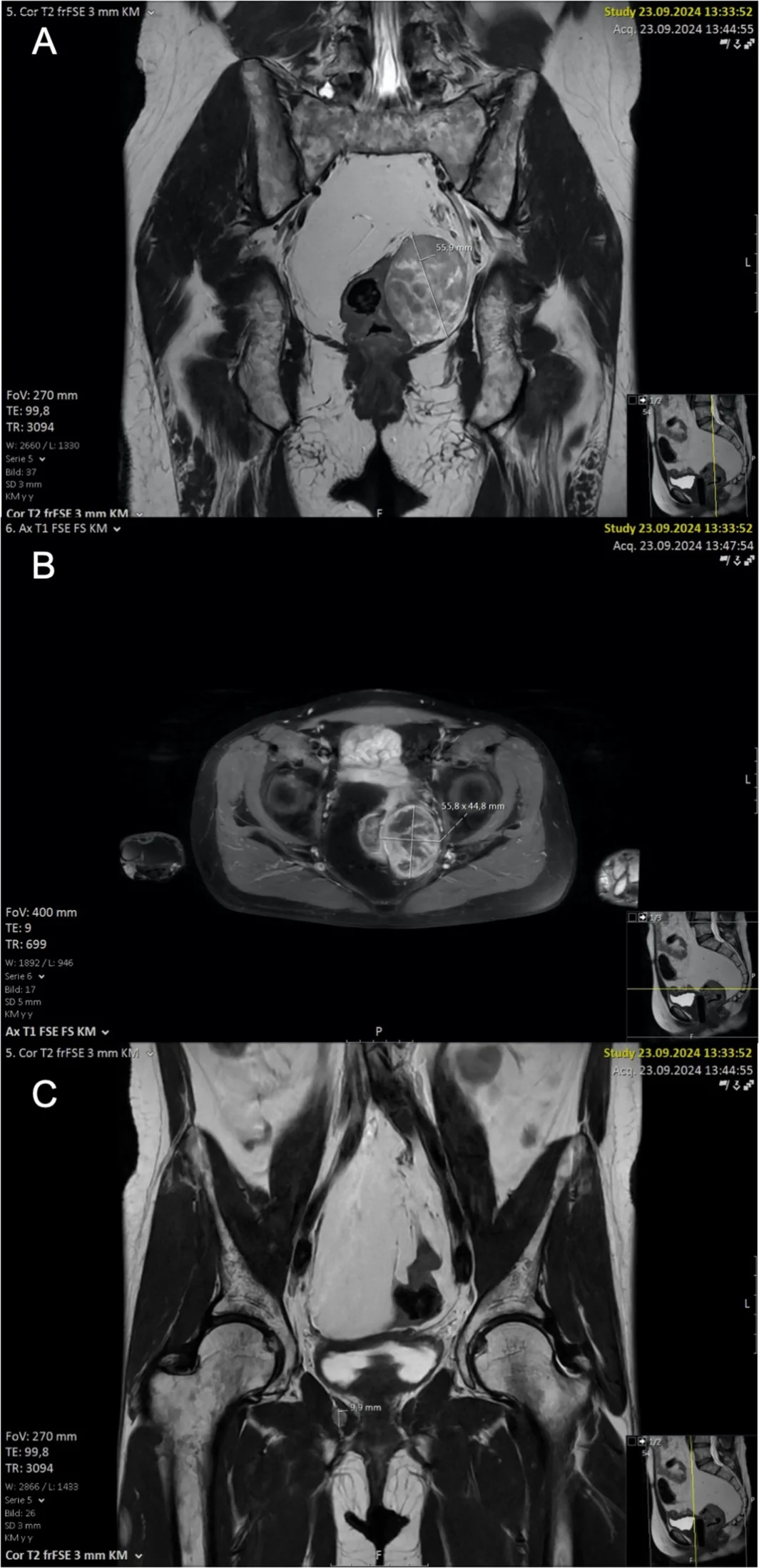
Pelvic MRI native and contrast‐enhanced 1 year post‐total‐hysterectomy. (A) Coronal plane. A well‐circumscribed mass is identified in the left lateral pararectal region. The lesion demonstrates heterogeneous T2‐weighted intermediate to high signal intensity with heterogeneous contrast enhancement. Broad‐based contact is present with mild impression upon the mid‐rectal segment. (B) Axial plane. T1‐weighted fast spin echo with fat suppression post‐contrast. The lesion shows heterogeneous contrast enhancement with hypointense areas. (C) Coronal plane. Anteriorly, at the level of the urethra, between the levator ani muscle (puborectalis part) and the obturator internus muscle, there is evidence of an additional well‐defined lesion measuring 11 × 7 × 9 mm, with intermediate signal intensity on this T2‐weighted image.

## 3 Differential Diagnosis, Investigations and Treatment

A laparoscopic operation was performed. During this operation, the rectum was found to be displaced to the right, and next to it, a 7 × 7 cm mass was revealed in the left pararectal space. The mass, which appeared partially degenerative and necrotic, was carefully enucleated (Figure 2). The suspected fibroid was separated from the surrounding tissue in toto and removed. It was retrieved using a retrieval bag and sent to pathology. In the next step, the plica medialis was transected approximately 2 cm above the bladder border cranially, and the incision was extended bilaterally. Subsequently, the vesico‐umbilical fascia and the Retzius space were opened down to the deep levator layer on the right side next to the urethra. This revealed a normal anatomy with no evidence of a mass. An intraoperative transvaginal ultrasound was performed to locate the paraurethral finding described preoperatively. This tumor was located below the endopelvic fascia below the levator layer. Therefore, further periurethral preparation from the abdominal cavity was abandoned.

**FIGURE 2:**
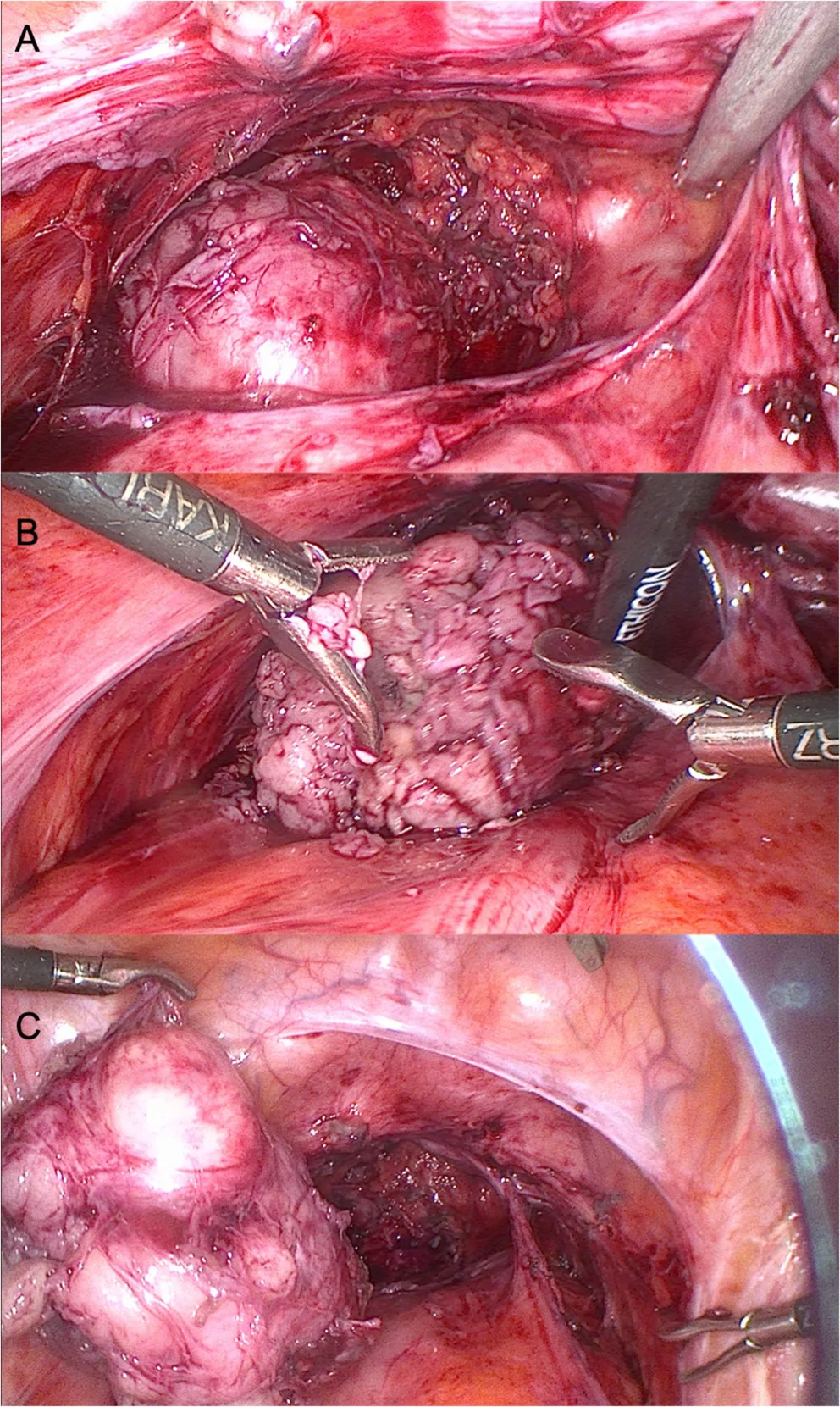
Intraoperative findings. (A) Following rectal mobilization, peritoneal division was performed on the left at the pelvic inlet level, along with uterolysis. This revealed a 7 × 7 cm space‐occupying lesion in the left pararectal area. (B) The lesion demonstrated partial degenerative and necrotic changes. The suspected myoma was separated from the surrounding tissue and entirely removed.

Postoperative pathological examinations raised suspicion of a malignant finding. Light microscopic examinations of the paraffin‐embedded tumor samples showed a partially necrotic tumor with spindle‐shaped, partly pleomorphic cells and focally highly pleomorphic, bizarre cell nuclei with up to 8 mitoses per 10 high‐power fields (HPFs). Immunohistochemically, the tumor was positive for actin, caldesmon, and partially desmin, and negative for pancytokeratin and S100. The proliferation rate (Ki‐67) was 30%. Molecular pathology FISH examination showed no evidence of MDM2 gene amplification. Taken together, these findings indicated elements of a leiomyosarcoma with an FNCLCC (Fédération Nationale des Centres de Lutte Contre le Cance) total‐ score [9] of 5, which was composed of tumor differentiation (3), mitotic count (1), and tumor necrosis (1) (corresponding to grade 2). While histopathology suggested a most likely incomplete (R1) resection, intraoperative findings were consistent with complete macroscopic tumor removal.

Given the now changed prognosis, a whole‐body positron emission tomography (PET)‐CT with 150 MBq 18‐F‐FDG was performed for further staging, as recommended by our hospital's gynecological tumor board. This showed no evidence of suspicious lymph nodes or thoracoabdominal metastases, and no evidence of Fluoro‐deoxyglucose (FDG)‐positive, malignancy‐suspicious focal lesions. A slightly increased FDG uptake in the left pararectal area was assessed as primarily postoperative activity. The incidental paraurethral mass proved to be FDG‐negative. This yielded a stage of T2bN0M0, according to the Fédération Internationale de Gynécologie et d'Obstétrique (FIGO) classification stage IIB [6, 10]. Assuming a localized disease, the interdisciplinary oncological tumor board initially recommended adjuvant systemic therapy with four cycles of doxorubicin and dacarbacin (DTIC). After the patient's presentation to internal medicine, however, this recommendation was revised, as the high previous exposure to anthracyclines from the prior therapies had to be taken into account, considering the resulting increased risk of cardiotoxicity. Finally, a recommendation for structured follow‐up was made.

## 4 Conclusion and Results (Outcome and Follow‐Up)

Due to a previous exposure to anthracyclines for the treatment of prior malignancies follow‐ up care alone was recommended. Regarding the FDG‐negative paraurethral mass a follow‐up MRI after 3 months was scheduled. The mass remained size‐stable and was managed conservatively with imaging surveillance without biopsy.

This rare case of a pararectal leiomyosarcoma occurring 1 year after total hysterectomy without morcellation illustrates the diagnostic challenges of smooth muscle tumors and the possibility of extrauterine disease manifestation. As we currently lack a recommended monitoring strategy for fibroids in reproductive‐aged women, with or without hysterectomy, this case emphasizes the importance of careful clinical and histopathological evaluation when new pelvic masses occur. Further reports are needed to clarify potential mechanisms and guide future management.

## 5 Discussion

Considering the presented case there is an ongoing discussion of how to distinguish between a benign leiomyoma and a malignant uterine sarcoma before organ‐preserving therapy such as myomectomy or before using morcellation during hysterectomy. The diagnosis of uterine leiomyosarcoma is frequently established only after surgical procedures that were initially performed under the assumption of benign leiomyomas. This circumstance highlights a major challenge in the surgical management of presumed fibroids. Laparoscopic myomectomy provides comparable safety and fertility outcomes to open surgery while reducing perioperative morbidity [11] but when an occult sarcoma is inadvertently operated on as a benign lesion, there is a substantial risk of intraoperative dissemination of malignant cells, worsening the patient's prognosis [12]. As described before, a preoperative assessment is clinically very challenging. The main debate on surgical approaches primarily focuses on the presence of sarcoma diagnoses in the initial tumor pathology [11]. However, regarding the prevalence of delayed sarcoma diagnoses after such procedures, clinical information is rare. Even less is known about the occurrence of leiomyosarcomas after total hysterectomy without morcellation.

One can find a case report describing the occurrence of a leiomyosarcoma 4 years after laparoscopic‐assisted supracervical hysterectomy (LASH) in combination with morcellation for uterine myomas [13]. It should be emphasized that the subsequently developed uterine sarcoma was intraoperatively adherent to the remaining cervical tissue, suggesting that the tumor may have originated from this tissue.

Additionally, a large study by Tan‐Kim et al. showed that in 3 out of 941 (0.3%) examined women with a history of hysterectomy combined with power morcellation, a uterine sarcoma was diagnosed with delay, after the patients were initially diagnosed with benign leiomyoma during the primary operation [2]. Upon final pathological evaluation, these malignancies were histologically identified as endometrial stromal sarcoma in one case and leiomyosarcomas in two cases. The median time between primary and secondary operations for these patients was 6 years. In one patient, reevaluation of the primary morcellated tissue sample revealed that a low‐grade endometrial stromal sarcoma was already present during the initial operation [2]. This case illustrates another problem with morcellation: the fragmentation of the primary tumor mass significantly complicates pathological evaluation of these tissue samples.

The same study also examined the frequency of uterine sarcomas in patients who underwent hysterectomy without morcellation. In this group, 5/2582 (0.19%) women received a diagnosis of uterine sarcoma based on the initially removed tumor tissue. No case of delayed sarcoma diagnosis after initial benign findings was reported in this group [2]. This study therefore underlines the utmost rarity of our case.

Burde et al. reported a case of a leiomyosarcoma occurring in a 50‐year‐old patient approximately 3 years after transabdominal hysterectomy with bilateral adnexectomy. As in our case, the initial operation yielded a histological diagnosis of leiomyoma; however, later reevaluation of the initially removed primary tumor revealed a diagnosis of a STUMP (Smooth Muscle Tumor of Uncertain Malignant Potential) [14].

The case presented in this report is most comparable to the latter case, as total hysterectomy was performed in both cases. The question arises regarding which pathophysiological mechanisms could underlie the occurrence of a parasitic uterine sarcoma after total hysterectomy. While one might consider the dispersal of myoma cells during morcellation that could later transform into sarcoma cells [15] or the dispersal of already initially transformed cells that were overlooked due to complicated pathological diagnosis, this explanatory approach is difficult to imagine in cases following total hysterectomy without morcellation.

In our case repeat histopathological review of the initial intramural fibroids was not pursued due to unequivocally benign initial findings, representing a limitation of this case and underscoring the diagnostic challenge of distinguishing parasitic transformation from metastasis in rare leiomyosarcoma presentations.

A possible explanation in our case could be a small parasitic pararectal myoma that had already existed during the initial surgery and eventually transformed [16], or an unrelated leiomyosarcoma developed in the pararectal peritoneum. The development of such a rare tumor may have been facilitated by prior exposure to anthracycline‐based chemotherapy or by an underlying genetic predisposition to tumorigenesis [1]. Although a direct causal association between BRCA1 alterations and smooth muscle tumors has not been firmly established, BRCA1 plays a central role in DNA damage repair and genomic stability. Defects in homologous recombination pathways have been implicated in the pathogenesis of uterine sarcomas [6], suggesting a potential mechanistic link in rare cases. In patients with known BRCA1 alterations, particularly mosaic variants, the occurrence of atypical mesenchymal malignancies may warrant heightened clinical awareness and supports the concept of individualized surveillance beyond organ‐specific cancer risks.

## Author Contributions

**Nanna Monjé:** conceptualization, investigation, visualization, writing – original draft. **Matthias Alexa:** conceptualization, methodology. **Annette Hasenburg:** resources, supervision. **Yaman Degirmenci:** conceptualization, investigation, methodology, project administration, resources, supervision.

## Funding

The authors have nothing to report.

## Ethics Statement

The authors have nothing to report.

## Consent

Written informed consent was obtained from the patient for the publication of this case report.

## Conflicts of Interest

The authors declare no conflicts of interest.

## Data Availability

The data that support the findings of this study are available on request from the corresponding author. The data are not publicly available due to privacy or ethical restrictions.
